# 4-Bromo-*N*-(4-bromo­phen­yl)aniline

**DOI:** 10.1107/S160053681100715X

**Published:** 2011-03-02

**Authors:** Michelle M. Duong, Joseph M. Tanski

**Affiliations:** a Department of Chemistry, Vassar College, Poughkeepsie, NY 12604, USA

## Abstract

In the title compound, C_12_H_9_Br_2_N, the dihedral angle between the benzene rings is 47.32 (5)°, whereas the pitch angles, or the angles between the mean plane of each aryl group ‘propeller blade’ and the plane defined by the aryl bridging C—N—C angle, are 18.1 (2) and 31.7 (2)°. No inter­molecular N—H hydrogen bonding is present in the crystal; however, there is a short inter­molecular Br⋯Br contact of 3.568 (1) Å.

## Related literature

The title compound is an amine analogue of brominated diphenyl ether flame retardant materials commonly used in household items. For information on environmental and health concerns related to brominated flame retardants, see: de Wit (2002) ▶; Lunder *et al.* (2010 ▶). For the synthesis of the title compound, see: Crounse & Raiford (1945 ▶); Galatis & Megaloikonomos (1934 ▶); He *et al.* (2008 ▶). For related structures, see: Eriksson *et al.* (2004 ▶); Plieth & Ruban (1961 ▶); Li *et al.* (2010 ▶). For the van der Waals radius of Br and inter­molecular Br⋯Br contacts, see: Bondi (1964 ▶); Medlycott *et al.* (2007 ▶). For a description of the pitch angle, see: Lim & Tanski (2007 ▶).
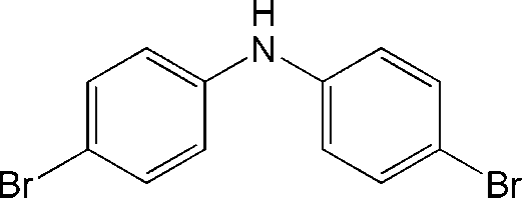


## Experimental

### 

#### Crystal data

C_12_H_9_Br_2_N
*M* *_r_* = 327.02
Monoclinic, 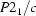

*a* = 5.9993 (12) Å
*b* = 13.032 (3) Å
*c* = 14.228 (3) Å
β = 96.967 (3)°
*V* = 1104.2 (4) Å^3^
*Z* = 4
Mo *K*α radiation
μ = 7.30 mm^−1^
*T* = 125 K
0.30 × 0.30 × 0.17 mm

#### Data collection

Bruker APEXII CCD diffractometer
Absorption correction: multi-scan (*SADABS*; Bruker 2007 ▶) *T*_min_ = 0.218, *T*_max_ = 0.370
17275 measured reflections
3373 independent reflections
2786 reflections with *I* > 2σ(*I*)
*R*_int_ = 0.038

#### Refinement

*R*[*F* ^2^ > 2σ(*F* ^2^)] = 0.026
*wR*(*F* ^2^) = 0.064
*S* = 1.02
3373 reflections
139 parameters
1 restraint
H atoms treated by a mixture of independent and constrained refinement
Δρ_max_ = 0.94 e Å^−3^
Δρ_min_ = −0.45 e Å^−3^

### 

Data collection: *APEX2* (Bruker, 2007 ▶); cell refinement: *SAINT* (Bruker, 2007 ▶); data reduction: *SAINT*; program(s) used to solve structure: *SHELXS97* (Sheldrick, 2008 ▶); program(s) used to refine structure: *SHELXL97* (Sheldrick, 2008 ▶); molecular graphics: *SHELXTL* (Sheldrick, 2008 ▶); software used to prepare material for publication: *SHELXTL*.

## Supplementary Material

Crystal structure: contains datablocks I, global. DOI: 10.1107/S160053681100715X/si2339sup1.cif

Structure factors: contains datablocks I. DOI: 10.1107/S160053681100715X/si2339Isup2.hkl

Additional supplementary materials:  crystallographic information; 3D view; checkCIF report

## Figures and Tables

**Fig. 1.**
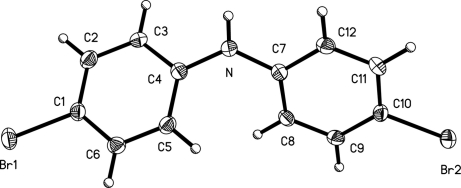
A view of compound (I), with displacement ellipsoids shown at the 50% probability level.

## supplementary crystallographic information

### Comment

The title compound, 4-bromo-*N*-(4-bromophenyl)aniline, C_12_H_9_Br_2_N (I), was first synthesized by Galatis & Megaloikonomos (1934) *via* the direct bromination of diphenylamine, and the structure was corroborated by Crounse & Raiford (1945) in their study of the hydrolysis of the benzoyl derivative. More recently, halogenated diphenylamines have been prepared by copper catalyzed coupling reactions (He *et al.*, 2008). The crystal structure of the chloride analogue is known (Plieth & Ruban, 1961), and an analogous structure with an oxygen bridge has also been reported (Eriksson *et al.*, 2004). The title compound is an amine analogues of a class of brominated diphenyl ether materials (de Wit, 2002). Polybrominated diphenyl ethers are commonly used as flame retardants (Eriksson *et al.*, 2004) in consumer products and electronics and have been found in humans (Lunder *et al.*, 2010).

Compound (I) is a dibrominated diphenyl amine derivative with a "propeller blade" disposition of the benzene rings about the bridging nitrogen atom. The structure reveals that there is no intermolecular hydrogen bonding, although there are significant intermolecular Br···Br contacts (Medlycott *et al.* , 2007) at a distance of 3.568 (1) Å, which is shorter than the sum of the van der Waals radius of bromine, 1.85Å (Bondi, 1964), at 3.7 Å. The aryl-bridging C4—N—C7 angle in (I) is 128.5 (2)°, somewhat smaller than the C—N—C bond angle of 133.8° found in the isomorphous dichloro analog (Plieth & Ruban, 1961), but similar to the C—N—C bond angle of 128.1° in another similar structure, *N*-4-(bromophenyl)-4-nitroaniline, which contains one bromo and one nitro group (Li *et al.*, 2010).

The dihedral angle in (I) is found to be 47.32 (5)°, whereas the pitch angles are 18.1 (2)° and 31.7 (2)°. The pitch angles are the angles between the mean plane of each aryl group "propeller blade" and the plane defined by the aryl bridging C4—N—C7 angle. The pitch angles are metrical parameters that describe the dispostion of the benzene rings about the bridging atom with greater detail than the dihedral angle; structures with equivalent dihedral angles may exhibit dramatically different orientations of the benzene rings about the bridging group (Lim & Tanski, 2007). In the isomorphous dichloro analog to the title compound, the dihedral angle is found to be significantly larger, 56.5°, as are the pitch angles of 22.1° and 39.1°. In another similar bromo compound, *N*-4-(bromophenyl)-4-nitroaniline, where the dihedral angle of 44.8° is more similar to that of the title compound, the pitch angles are found to be 12.6° and 35.1°.

### Experimental

Crystalline 4-bromo-*N*-(4-bromophenyl)aniline (I) was purchase from Aldrich Chemical Company, USA.

### Refinement

All non-hydrogen atoms were refined anisotropically. The hydrogen atoms on carbon were included in calculated positions and were refined using a riding model at C–H = 0.95Å and *U*_iso_(H) = 1.2 × *U*_eq_(C) of the aryl C-atoms. T hydrogen atom on nitrogen was refined semifreely with the help of a distance restraint, d(N–H) = 0.835 (16) Å and *U*_iso_(H) = 1.2 × *U*_eq_(N). The extinction parameter (EXTI) refined to zero and was removed from the refinement.

### Crystal data

**Table:**

C_12_H_9_Br_2_N	*F*(000) = 632
*M**_r_* = 327.02	*D*_x_ = 1.967 Mg m^−^^3^
Monoclinic, *P*2_1_/*c*	Mo *K*α radiation, λ = 0.71073 Å
Hall symbol: -P 2ybc	Cell parameters from 8371 reflections
*a* = 5.9993 (12) Å	θ = 2.9–30.4°
*b* = 13.032 (3) Å	µ = 7.30 mm^−^^1^
*c* = 14.228 (3) Å	*T* = 125 K
β = 96.967 (3)°	Plate, colourless
*V* = 1104.2 (4) Å^3^	0.30 × 0.30 × 0.17 mm
*Z* = 4	

### Data collection

**Table:**

Bruker APEXII CCD diffractometer	3373 independent reflections
Radiation source: fine-focus sealed tube	2786 reflections with *I* > 2σ(*I*)
graphite	*R*_int_ = 0.038
φ and ω scans	θ_max_ = 30.6°, θ_min_ = 2.1°
Absorption correction: multi-scan (*SADABS*; Bruker 2007)	*h* = −8→8
*T*_min_ = 0.218, *T*_max_ = 0.370	*k* = −18→18
17275 measured reflections	*l* = −20→20

### Refinement

**Table:**

Refinement on *F*^2^	Primary atom site location: structure-invariant direct methods
Least-squares matrix: full	Secondary atom site location: difference Fourier map
*R*[*F*^2^ > 2σ(*F*^2^)] = 0.026	Hydrogen site location: inferred from neighbouring sites
*wR*(*F*^2^) = 0.064	H atoms treated by a mixture of independent and constrained refinement
*S* = 1.02	*w* = 1/[σ^2^(*F*_o_^2^) + (0.0365*P*)^2^] where *P* = (*F*_o_^2^ + 2*F*_c_^2^)/3
3373 reflections	(Δ/σ)_max_ = 0.001
139 parameters	Δρ_max_ = 0.94 e Å^−^^3^
1 restraint	Δρ_min_ = −0.45 e Å^−^^3^

### Special details

**Table:**

Experimental. A suitable crystal was mounted in a nylon loop with Paratone-N cryoprotectant oil and data was collected on a Bruker *APEX* 2 CCD platform diffractometer. The structure was solved using direct methods and standard difference map techniques, and was refined by full-matrix least-squares procedures on F^2^ with *SHELXTL* Version 6.14 (Sheldrick, 2008).
Geometry. All e.s.d.'s (except the e.s.d. in the dihedral angle between two l.s. planes) are estimated using the full covariance matrix. The cell e.s.d.'s are taken into account individually in the estimation of e.s.d.'s in distances, angles and torsion angles; correlations between e.s.d.'s in cell parameters are only used when they are defined by crystal symmetry. An approximate (isotropic) treatment of cell e.s.d.'s is used for estimating e.s.d.'s involving l.s. planes.
Refinement. Refinement of *F*^2^ against ALL reflections. The weighted *R*-factor *wR* and goodness of fit *S* are based on *F*^2^, conventional *R*-factors *R* are based on *F*, with *F* set to zero for negative *F*^2^. The threshold expression of *F*^2^ > σ(*F*^2^) is used only for calculating *R*-factors(gt) *etc*. and is not relevant to the choice of reflections for refinement. *R*-factors based on *F*^2^ are statistically about twice as large as those based on *F*, and *R*- factors based on ALL data will be even larger.

### Fractional atomic coordinates and isotropic or equivalent isotropic displacement parameters (Å^2^)

**Table:**

	*x*	*y*	*z*	*U*_iso_*/*U*_eq_	
Br1	0.35642 (4)	0.125752 (16)	0.143618 (15)	0.02649 (7)	
Br2	−0.19656 (3)	0.946875 (15)	0.134200 (15)	0.02372 (7)	
N	0.4407 (3)	0.58823 (13)	0.10486 (13)	0.0224 (4)	
H1	0.566 (3)	0.6043 (18)	0.0893 (16)	0.027*	
C1	0.3790 (3)	0.27103 (16)	0.13498 (13)	0.0200 (4)	
C2	0.5839 (3)	0.31866 (15)	0.16241 (14)	0.0229 (4)	
H2A	0.7114	0.2793	0.1865	0.027*	
C3	0.5998 (3)	0.42427 (15)	0.15413 (14)	0.0215 (4)	
H3A	0.7391	0.4573	0.1733	0.026*	
C4	0.4131 (3)	0.48316 (15)	0.11792 (13)	0.0186 (4)	
C5	0.2092 (3)	0.43296 (15)	0.09209 (14)	0.0204 (4)	
H5A	0.0806	0.4719	0.0685	0.025*	
C6	0.1911 (3)	0.32763 (15)	0.10030 (14)	0.0209 (4)	
H6A	0.0514	0.2944	0.0824	0.025*	
C7	0.2844 (3)	0.66694 (15)	0.10980 (14)	0.0188 (4)	
C8	0.0933 (3)	0.65683 (15)	0.15638 (14)	0.0192 (4)	
H8A	0.0621	0.5931	0.1845	0.023*	
C9	−0.0513 (3)	0.73940 (15)	0.16173 (13)	0.0194 (4)	
H9A	−0.1822	0.7318	0.1925	0.023*	
C10	−0.0044 (3)	0.83266 (14)	0.12218 (14)	0.0191 (4)	
C11	0.1859 (3)	0.84517 (15)	0.07631 (14)	0.0208 (4)	
H11A	0.2179	0.9096	0.0497	0.025*	
C12	0.3281 (3)	0.76224 (15)	0.06992 (13)	0.0202 (4)	
H12A	0.4572	0.7701	0.0380	0.024*	

### Atomic displacement parameters (Å^2^)

**Table:**

	*U*^11^	*U*^22^	*U*^33^	*U*^12^	*U*^13^	*U*^23^
Br1	0.03250 (12)	0.01599 (11)	0.03174 (12)	0.00176 (8)	0.00695 (9)	−0.00159 (8)
Br2	0.02408 (11)	0.01647 (10)	0.03091 (12)	0.00214 (7)	0.00456 (8)	−0.00193 (7)
N	0.0179 (8)	0.0174 (8)	0.0330 (10)	0.0004 (7)	0.0076 (7)	0.0013 (7)
C1	0.0219 (9)	0.0166 (9)	0.0217 (9)	0.0016 (7)	0.0039 (7)	−0.0020 (7)
C2	0.0197 (9)	0.0232 (10)	0.0253 (10)	0.0046 (8)	0.0008 (8)	0.0007 (8)
C3	0.0144 (9)	0.0215 (10)	0.0283 (10)	−0.0001 (7)	0.0012 (7)	−0.0016 (8)
C4	0.0203 (9)	0.0182 (9)	0.0180 (9)	−0.0002 (7)	0.0051 (7)	−0.0009 (7)
C5	0.0184 (9)	0.0210 (10)	0.0212 (9)	0.0031 (7)	−0.0006 (7)	−0.0014 (7)
C6	0.0189 (9)	0.0225 (10)	0.0211 (9)	−0.0008 (7)	0.0020 (7)	−0.0033 (7)
C7	0.0192 (9)	0.0167 (9)	0.0205 (9)	−0.0008 (7)	0.0019 (7)	0.0001 (7)
C8	0.0204 (9)	0.0155 (9)	0.0219 (9)	−0.0030 (7)	0.0038 (7)	0.0002 (7)
C9	0.0176 (9)	0.0207 (10)	0.0206 (9)	−0.0031 (7)	0.0049 (7)	−0.0008 (7)
C10	0.0192 (9)	0.0150 (9)	0.0227 (9)	0.0017 (7)	0.0005 (7)	−0.0019 (7)
C11	0.0239 (10)	0.0156 (9)	0.0232 (10)	−0.0042 (7)	0.0038 (8)	0.0009 (7)
C12	0.0193 (9)	0.0210 (9)	0.0210 (9)	−0.0027 (7)	0.0049 (7)	−0.0007 (7)

### Geometric parameters (Å, °)

**Table:**

Br1—C1	1.903 (2)	C5—H5A	0.9500
Br2—C10	1.9031 (19)	C6—H6A	0.9500
N—C4	1.394 (3)	C7—C8	1.399 (3)
N—C7	1.397 (3)	C7—C12	1.403 (3)
N—H1	0.835 (16)	C8—C9	1.390 (3)
C1—C6	1.387 (3)	C8—H8A	0.9500
C1—C2	1.390 (3)	C9—C10	1.383 (3)
C2—C3	1.386 (3)	C9—H9A	0.9500
C2—H2A	0.9500	C10—C11	1.391 (3)
C3—C4	1.403 (3)	C11—C12	1.387 (3)
C3—H3A	0.9500	C11—H11A	0.9500
C4—C5	1.397 (3)	C12—H12A	0.9500
C5—C6	1.383 (3)		
			
C4—N—C7	128.53 (17)	C1—C6—H6A	120.4
C4—N—H1	114.0 (17)	N—C7—C8	123.26 (17)
C7—N—H1	117.4 (17)	N—C7—C12	118.05 (17)
C6—C1—C2	121.03 (19)	C8—C7—C12	118.62 (18)
C6—C1—Br1	119.41 (15)	C9—C8—C7	120.40 (18)
C2—C1—Br1	119.56 (15)	C9—C8—H8A	119.8
C3—C2—C1	119.14 (18)	C7—C8—H8A	119.8
C3—C2—H2A	120.4	C10—C9—C8	119.93 (18)
C1—C2—H2A	120.4	C10—C9—H9A	120.0
C2—C3—C4	120.97 (18)	C8—C9—H9A	120.0
C2—C3—H3A	119.5	C9—C10—C11	120.87 (18)
C4—C3—H3A	119.5	C9—C10—Br2	119.70 (15)
N—C4—C5	122.64 (18)	C11—C10—Br2	119.40 (14)
N—C4—C3	118.93 (17)	C12—C11—C10	119.05 (18)
C5—C4—C3	118.36 (18)	C12—C11—H11A	120.5
C6—C5—C4	121.20 (18)	C10—C11—H11A	120.5
C6—C5—H5A	119.4	C11—C12—C7	121.13 (18)
C4—C5—H5A	119.4	C11—C12—H12A	119.4
C5—C6—C1	119.28 (19)	C7—C12—H12A	119.4
C5—C6—H6A	120.4		
			
C6—C1—C2—C3	−0.5 (3)	C4—N—C7—C8	20.0 (3)
Br1—C1—C2—C3	178.75 (15)	C4—N—C7—C12	−163.25 (19)
C1—C2—C3—C4	−0.5 (3)	N—C7—C8—C9	177.59 (18)
C7—N—C4—C5	33.3 (3)	C12—C7—C8—C9	0.8 (3)
C7—N—C4—C3	−149.9 (2)	C7—C8—C9—C10	−1.1 (3)
C2—C3—C4—N	−175.67 (18)	C8—C9—C10—C11	0.4 (3)
C2—C3—C4—C5	1.3 (3)	C8—C9—C10—Br2	−177.49 (14)
N—C4—C5—C6	175.75 (18)	C9—C10—C11—C12	0.5 (3)
C3—C4—C5—C6	−1.1 (3)	Br2—C10—C11—C12	178.40 (15)
C4—C5—C6—C1	0.1 (3)	C10—C11—C12—C7	−0.8 (3)
C2—C1—C6—C5	0.7 (3)	N—C7—C12—C11	−176.84 (18)
Br1—C1—C6—C5	−178.55 (14)	C8—C7—C12—C11	0.1 (3)
